# The Mechanism of the Ossicles of the Ear

**Published:** 1874-02

**Authors:** 


*The Mechanism of the Ossicles of the Ear and Membrana Tympani.* By H. Helmholtz. Translated from the German by Albert H. Buck and Normand Smith. With Twelve Illustrations. New York: D. Appleton & Co., 1873. Buffalo: Martin Taylor.

This essay attempts to place in a clearer light, and to demonstrate more perfectly than has hitherto been done, the problems concerning the action of the conducting apparatus of the ear in transmitting sound.

Prof. Helmholtz, is a thorough and careful investigator, and this work will be received by those interested in the department of otology with pleasure. The translation is well made, although from the terse style of the author such a task is far from easy. The eight sections into which the work is divided, treat of the results due to the small dimensions of the auditory apparatus, the Anatomy of the Membrana Tympani, the attachments of the Hammer, and of the Anvil; the movements of the Stirrup, the concerted action of the bones of the ear, the Mechanism of the Membrana Tympani, and a mathamatical consideration of the mechanism of curved membranes. As a scientific work this essay deserves a first position, and the greatest regret which we have, is that it will be read and appreciated by but few, except those who make the study of otology a special work.

